# From Evidence to Practice: Quilting for Seroma Prevention After Mastectomy and Surgeons' Acceptance

**DOI:** 10.7759/cureus.96494

**Published:** 2025-11-10

**Authors:** Dua Hashmi, Cho Ee Ng, Rasheed Elayyan, Tarannum Fasih

**Affiliations:** 1 General Surgery, Whipps Cross Hospital, Barts Health NHS Trust, London, GBR; 2 General Surgery, Royal Liverpool University Hospital, NHS University Hospitals of Liverpool Group, Liverpool, GBR; 3 Breast Surgery, Queen Elizabeth Hospital, Gateshead Health NHS Foundation Trust, Gateshead, GBR

**Keywords:** breast cancer, flap fixation, mastectomy, mastectomy flap, patient comfort, quilting, seroma

## Abstract

Introduction

Seroma formation is a frequent complication after simple mastectomy due to the creation of a large dead space. Multiple aspirations are required to manage patients' symptoms. At present, breast surgeons lack consensus on effective preventative strategies. To address this issue, we have implemented a technique called quilting, which involves suturing the mastectomy flap to the chest wall muscle to convert the dead space into smaller compartments, thereby reducing the possibility of seroma.

Method

An audit was carried out from April 2020 to June 2021 to establish postoperative seroma rates for patients undergoing simple mastectomy without reconstruction. A subsequent evaluation was completed between October 2023 and December 2024 following the adoption of quilting as standard practice. After each stage, results were shared with UK breast surgeons along with a survey to assess perceptions of quilting and other preventive strategies. An analysis was then done to examine the relationship between sharing audit data and the decision to quilt.

This study seeks to outline the effectiveness of quilting in the prevention of seroma formation while also assessing current and alternative practices of UK surgeons in managing seromas. The insights gained from the quilting audit were shared in a follow-up survey to challenge the perception surgeons held of quilting and to consider incorporating this technique into their future practices.

Results

A total of 66 simple mastectomies were performed, with 32 patients receiving quilting and 34 undergoing conventional closure. On average, each patient in the quilting group received 13 sutures, taking an average of nine minutes for suturing. Among the 32 patients in the quilting group, there were no failed day cases due to postoperative haematomas or complications such as pain, wound breakdown, or long-term disfigurement of the flap. Only one case developed a seroma, but the volume was not clinically significant enough to require drainage. The non-quilting group showed a 70% seroma rate, with patients averaging three aspirations each, averaging 230 ml of fluid.

Sixty UK-based surgeons responded to the first survey and 48 to the second. The first survey revealed that only 11.6% of surgeons offered quilting, 48% relied on drains, and 35% took no measures to prevent seroma formation. After sharing the quilting audit results, 79% of respondents indicated they would consider implementing quilting, while 20% remained resistant to the idea. The changes to response were statistically significant (p<0.00001).

Conclusion

The feedback from the surveys indicates a lack of consensus among UK surgeons regarding the prevention of seromas post-mastectomy. Nonetheless, there is a significant willingness to explore quilting. Our findings demonstrate that quilting is both safe and effective in reducing seroma formation, leading to improved patient outcomes. We recommend that future research focus on establishing comprehensive national evidence and guidelines aimed at decreasing seroma rates, thus addressing the current variability in approach across the country.

## Introduction

Mastectomy is performed in 40% of breast cancer cases in the United Kingdom [1]. Not all patients undergo immediate reconstruction following a simple mastectomy. The most common complication after a simple mastectomy is seroma collection beneath the mastectomy flap [2,3]. A clinically significant seroma (CSS) is identified [4] when it necessitates multiple aspirations to alleviate pain and manage symptoms. This is burdensome for patients, who must make frequent trips to the hospital and may experience delays to adjuvant treatment, including radiotherapy and chemotherapy [1]. The use of drains has been one of the most common practices among breast surgeons [5,6]. Alternatives include the application of glue, compression, and quilting of the mastectomy flap [2,3,7]. However, these are less commonly used and often depend on the surgeon's preference and willingness to address the issue.

We completed a closed-loop audit. The first part reviewed mastectomy cases from April 2020 to June 2021, when simple closure without quilting was standard practice; the subsequent part reviewed mastectomies from October 2023 to December 2024, when quilting had become standard practice for all mastectomy cases in our unit. The need for a change in practice was identified following a high incidence of CSS. This prompted us to introduce quilting for the mastectomy flap. During the initial learning and refinement phase (July 2021 to September 2023), over 60 mastectomies were performed, but prospective data were not collected. Recognising the importance of this procedure, we subsequently developed the process to build on our earlier findings. This included additional information, such as the length of time for quilting, the number of sutures used, and the types of sutures utilised.

Due to the lack of consensus on effective preventative measures, surgeons currently operate without standardised recommendations or guidance and rely largely on their own experience and beliefs to manage CSS formation.

Aim

The objective of this study is twofold: first, to evaluate whether quilting has a positive impact on rates of seroma formation compared with conventional closure and, second, to describe a technique that can be standardised during mastectomy to prevent seroma formation. We also conducted a two-part survey among UK breast surgeons. The survey explored how they currently prevent and manage post-mastectomy seroma, their perception of quilting, and their willingness to adopt quilting based on the results of our audit.

This audit was previously presented as a poster presentation at the Royal College of Surgeons of Edinburgh (RCSEd) 23rd Annual QI & Audit Symposium, Edinburgh, on March 21, 2025.

## Materials and methods

We conducted a complete audit cycle that reviewed the outcomes of mastectomy procedures at Queen Elizabeth Hospital, Gateshead, England. The first part is from April 2020 to June 2021, and the second part is from October 2023 to December 2024. This allowed us to compare outcomes of seroma formation when using quilting versus when not.

Details of the data collected are shown in Table 1.

**Table 1:** Details of patient demographics and operative findings collected from patient records ASA: American Society of Anesthesiologists; BMI: body mass index

Patient data	
ASA grade	
BMI	
Smoking status	
Alcohol consumption habits	
History of diabetes	
History of previous breast surgery + radiotherapy treatment	
Procedural information	
Operative findings	
Antibiotic use and duration	
Use of pectoral block	
Surface area of the mastectomy bed	Height
	Width
Number of sutures used to quilt	
Weight of the breast removed	
Axillary procedure	None
	Sentinel lymph node biopsy
	Targeted clearance
	Formal clearance
Post-procedure	
Postoperative haematoma and return to theatre	
Failed day case	
Drain use	

The second part of the study involved two surveys sent to surgeons in the United Kingdom: one survey was distributed before the audit, and the other was sent following, including the results of the audit.

All surgeons, from surgical trainees (ST) to consultants, including specialty associate surgeons (SAS) who hold membership in the Association of Breast Surgery (ABS), were invited to participate in the survey. An online survey was created using Google Forms (Google LLC, Mountain View, California, United States) and was distributed to breast surgeons through the ABS forum. The survey was open from October 2024 for four weeks. E-mail reminders were sent to all members of the ABS. The second survey was sent in April 2025 to UK breast surgeons again through the ABS forum, now remaining open for eight weeks.

Both surveys inquired about the location in which the surgeons were currently employed, and the second survey also asked about their role. The questionnaire developed for this is included in the Appendices. 

Participants for quilting and non-quilting

All patients undergoing simple mastectomy with or without axillary procedures, including risk-reducing mastectomies, were included in the study. Any patient undergoing immediate reconstruction was excluded. 

Data was collected across patient records, theatre data, and operative notes. Clinical records and nursing notes were reviewed to track the number of clinic visits for seroma aspiration and other postoperative complications.

All patients in this audit were treated under one team to mitigate technical variation in quilting technique and bias. All the parameters used were matched across both groups.

Procedure

All patients in both groups received intravenous prophylactic antibiotics using flucloxacillin 1 g at induction, and those with penicillin allergy received 400 mg of teicoplanin. They also all received a pectoral block using 40 ml of 0.25% levobupivacaine.

Non-quilting group: Following mastectomy, conventional closure of the dermis and skin was performed using interrupted Monocryl for dermis and continuous Monocryl 3/0 for the skin. These patients had one drain left in situ in the mastectomy bed and a second drain in the axilla when clearance was performed. The patients were followed up five days following the operation to assess the wound and remove the drains, irrespective of the volume of output. Patients were given an open appointment to visit the breast clinic for assessment by the breast care nurse, should they have a concern that the seroma was recollecting and it caused them discomfort. After evaluation by the breast care nurses and confirmation of seroma collection, aspiration of the seroma was performed, and the amount of fluid aspirated was documented in the nursing record. Patients were then given future appointments for anticipated further aspirations of the seroma within a week's time, along with an open appointment if the patient developed any issues prior to that week.

Quilting group: Patient data and details of the key operative findings were collected as shown in Table 1. Interrupted stitches are placed to oppose the pectoralis major and the mastectomy flap using Vicryl 2-0 sutures, spaced approximately 3-4 cm apart. Measures are taken to avoid injuring blood vessels within the mastectomy flap. No drains are left in situ, unless axillary clearance was performed, in which case an axillary drain would be sited in the axilla. Patients were discharged after a same-day mastectomy. The length of quilting is timed by an unscrubbed staff member using a stopwatch, while the first assistant counts the number of stitches. Postoperative patients are scheduled for follow-up visits in the clinic on day 5 for a wound check and to remove the axillary drain. They had a further follow-up again on day 10 to discuss their results. Patients were given open access to visit the breast care nurse-led clinic if any wound-related issues occurred.

The primary postoperative endpoints include seroma formation and the failure of day cases due to pain or the need to return to the theatre for haematoma treatment. Secondary endpoints include wound breakdown, pain, infection, and disfigurement.

The chi-squared test of independence was performed to examine the relation between sharing audit data and the decision to quilt. 

## Results

The survey responses are from surgeons performing mastectomies regularly across the United Kingdom. The demographic breakdown of responders by UK National Health Service (NHS) regions is shown in Table 2.

**Table 2:** Geographical distribution of UK breast surgeons responding to Survey 1 and Survey 2 by UK NHS regions NHS: National Health Service

NHS regions	Survey 1 (total: 60)	Survey 2 (total: 48)
Northeast Region	19	21
Northwest Region	8	8
Yorkshire and the Humber	7	3
Merseyside	7	2
East Midlands	6	2
Scotland	6	2
London	2	3
East of England	1	2
West Midlands	1	1
Southwest Peninsula	1	0
Southeast Region (KSS)	1	2
Not disclosed	1	0
Ireland	0	2

Patient data is compared across both quilting and non-quilting groups below in Table 3, demonstrating that the groups have similar characteristics.

**Table 3:** Comparison of patient characteristics between quilting and non-quilting groups ASA: American Society of Anesthesiologists; BMI: body mass index; WLE: wide local excision; SLNB: sentinel lymph node biopsy

Characteristics	Non-quilting (n=34)	Quilting (n=32)
Mean age	66	62
Mean BMI	32	30
History of diabetes mellitus	5	5
History of hypertension	8	9
Current smoker	8	11
Mean ASA	3	3
Patients with previous radiation treatment/WLE	8	12
Mean weight of breast (g)	1076	1041
Axillary procedure		
None	9	11
SLNB	18	16
Targeted clearance	0	2
Formal clearance	7	3
Patients with neoadjuvant chemotherapy	3	0
Patients under prophylactic procedure	9	7

A table summarising the outcome of CSS in both the quilting and non-quilting groups is shown in Table 4. No interventions were required in the groups after quilting.

**Table 4:** Comparison of clinically significant seroma outcomes between quilting and non-quilting groups

	Non-quilting	Quilting
Number of patients	34	32
Seroma formation	24	1
Mean aspirate per patient	3	0
Mean volume aspirate per episode (ml)	230	0

For the group who received quilting, an average of 13 quilting stitches was performed per patient, taking an average time of nine minutes per patient. None of the 32 patients failed day cases, with no postoperative complications, including haematomas, pain, wound breakdown, infection, or flap disfigurement. Only one case was identified to have a seroma, but the volume was not adequate to be drained or cause the patient symptoms. 

The results of pre- and post-audit surveys are shown in Tables 5-6.

**Table 5:** Summary of findings from Survey 1

Survey 1: pre-audit respondents (60)	
Do you regularly perform mastectomies?	
Yes	60
No	0
What technique do you use to manage seroma formation?	
Quilting	7 (11.6%)
Drain	29 (48.39%)
Compression	39 (5%)
Nil	21 (35%)
If you use quilting, what is your technique?	
Interrupted	3
Continuous	1
Other:	
Self-locking	1
Unknown	1
If you do not use quilting, why?	
Unfamiliar	3%
Unconvinced	38%
Time-consuming	28%
Other	31%
Other reasons for not using quilting included the following: the potential for multiloculated seroma formation, increased postoperative pain, aesthetic deformity, and risk of haematoma	

**Table 6:** Summary of findings from Survey 2

Survey 2: post-audit respondents (48)	
Do you agree that quilting has a role to play in preventing seroma formation?	
Yes	37
No	6
Other:	
No experience	2
Not sure	1
More evidence required	1
Sometimes	1
Would the result of the audit (as described above) make you consider quilting in the future?	
Yes	27 (56%)
No	9 (18.75%)
Other:	
Possible	2
Already practice	6
Small number	2
Not sure	2
Would you consider quilting for selected cases?	
Yes	36
No	8
Other:	
Already do it	2
No response	2
If you would consider quilting, what made you change your mind?	
Audit results	15 (31%)
Clinical experience	23 (47.9%)
Other:	
No reply	6 (12.5%)
Maybe	1 (2%)
Already do it	3 (6.25%)
If you do not wish to offer quilting, please specify your reasons for that	
Not convinced of the technique	0
Not convinced by the audit results	5
Other:	
Larger study required	4
Pain/disfigurement	1
Bleeding	2
Time constraint	1
No experience	1

Table 7 demonstrates the change in opinion after the results from our audit were shared.

**Table 7:** The relation between sharing audit data and decision to quilt

	Consider or already quilting	Non-quilting/no response
Pre-audit (Survey 1)	7	53
Post-audit (Survey 2)	38	10

The chi-squared test of independence was performed to examine the relation between showing audit data and the decision to quilt. The relation between these variables was significant (x^2^(1,N=108)=49.9886; p<0.00001). Following the inclusion of audit findings in Survey 2, more responders were considering or already performing quilting as a technique to manage postoperative seroma formation.

## Discussion

This study is unique in having two aspects: the first is a local audit on the effectiveness of quilting in reducing seroma rates, and the second comprises two surveys reviewing surgeons' practices in seroma prevention and their response to our local audit results.

The first survey aimed to assess the extent of quilting practices, also known as flap fixation [8], and understand the reasons why it was not being utilised more frequently, as well as what measures surgeons take in daily practice to minimise seroma formation after mastectomy.

Suction drains were first introduced in 1947 for patients undergoing mastectomy to prevent fluid accumulation in the mastectomy bed [9]. They are still a common technique for addressing seroma to this day, despite no robust evidence supporting their effectiveness [7]. In our pre-audit survey, 48% of respondents reported using drains to manage seromas. However, with the increasing prevalence of day case mastectomies, this method can be inconvenient for patients and challenging to manage once they are discharged home. This can lead to psychological distress for patients who are recovering from surgery, as they must monitor the drain closely. If the drain is dislodged, loses suction, or causes concern about infection, patients must seek urgent medical support, which can cause further distress.

The other 38% of respondents in the pre-audit survey reported offering no interventions to prevent seromas, as they were not convinced by any of the current practices, citing a lack of evidence. This suggests that current clinical practice predominantly relies on surgeons' existing beliefs; thus, rather than poor experience of the quilting technique, there is still a lack of awareness.

Furthermore, repeated seroma aspiration poses an emotional burden on patients and carries risks, such as infection, persistent pain, and prolonged recovery [8,10]. Additionally, it is time-consuming for clinicians, who must dedicate clinical time to performing these aspirations. Given these challenges, there is a strong need to gather stronger evidence on preventing postoperative seroma formation after mastectomy. Such studies would provide much-needed insights and help establish standardised practices to ultimately improve both patient satisfaction and surgical outcomes.

Following the distribution of our audit results, feedback from the second survey was significantly more positive toward quilting. Seventy-three percent of respondents in the post-audit survey were consultants. Only six consultants reported practising the technique routinely. When asked if surgeons would consider using quilting in selected cases, 37 out of 48 respondents said yes. Among the 25 consultants who would consider quilting, 18 would consider it based on the audit results only. However, some concerns were raised about the small sample size, highlighting the need for larger studies to provide more robust evidence. Moreover, 10 out of 11 specialty trainees expressed their willingness to perform the technique based on audit or their clinical experience. Overall, trainees showed a strong interest in adopting the technique, emphasising the need to incorporate it as a standard practice during mastectomy procedures. This would allow trainees to become familiar with the procedure during their training and gain confidence before transitioning into consultant roles.

The grade of the participant was not ascertained in the pre-audit survey, which is a limitation. It is assumed that most participants were consultants, followed by ST and SAS. This assumption is supported by the first question, which inquired about their regular practice of quilting the flap, to which 100% reported that they did. Furthermore, the survey was redistributed by email as a reminder, but only to consultants.

When assessing the literature for a consensus in preventing seromas, we found a distinct lack of robust evidence. We identified one literature review that included two randomised controlled trials and six non-randomised studies, which were a combination of prospective and retrospective studies. They compared the postoperative outcomes of drains versus no drains, sealant versus non-sealant, and quilting versus non-quilting [1,2]. Unfortunately, the study was unable to draw any significant conclusions due to a considerable risk of bias [1].

A meta-analysis with 21 studies analysed quilting versus non-quilting [5]. Ten of these studies were randomised controlled trials, seven were prospective cohort studies, and four were retrospective studies. Three thousand four hundred seventy-three patients were split into two groups: one group received quilting of the flap, and the other group received no quilting. The conclusion of the meta-analysis showed that quilting was important to reduce the volume of drainage and the length of hospital stay. It also showed that quilting had similar complication rates when compared to conventional closure [5]. Another meta-analysis showed quilting had a lower seroma rate when compared with conventional closure of the mastectomy flap [6]. Again, there was no significant difference in the rates of postoperative complications such as haematoma, surgical site infection, or flap necrosis between the two groups [6].

A similar meta-analysis reviewed 12 studies, in which 986 patients underwent flap fixation and 901 did not [8]. Seroma formation was seen in 22.41% of quilted cases and 43.6% among non-quilted cases. Surgical site infection was comparable, with no statistical differences between the two groups.

Different surgeons have used various methods of quilting (interrupted or running sutures) and materials (Vicryl, polyglactin, or self-locking suture). However, due to limited evidence, no single technique or suture material has been proven superior. A study by van Zeelst et al. [4] described four different quilting techniques using running sutures with either Strattifix or Vicryl material. Four percent of cases quilted with Strattifix resulted in seroma formation, compared to 25-33% in the Vicryl 2-0 group. They compared this to other studies conducted by Almond et al. [11] and Purushotham et al. [12], where interrupted sutures were used without drains, resulting in seroma rates of 49% and 55%, respectively [4]. However, in our experience, the seroma rate with quilting is 3%, and the seromas were not clinically significant enough to require aspiration. Moreover, we found that using interrupted sutures is important to maintain a clear view of the operating field and to avoid injuring blood vessels within the flap. By comparison, another study by Foulon et al. [10] employed continuous sutures with absorbable polyfilament materials, reporting a clinically drainable seroma rate of 30.8%, along with an additional 17 minutes for quilting, in contrast to nine additional minutes and an average of 12 stitches in our study.

Furthermore, there is growing recognition of the need to tackle seroma formation. Several European trials have been completed or are in progress, aiming to provide evidence and validate the procedure of quilting. A Dutch trial called SAM included three arms: conventional closure, quilting, and the use of glue. The study demonstrated the benefit of quilting over the other methods. However, all patients in the trial had drains in place [13]. A Dutch single-centre survey known as the SARA Trial appears promising in evaluating the role of drains and in determining whether flap fixation alone is sufficient to reduce seroma formation after mastectomy [14-16]. Similarly, a French trial called QUISERMAS, which is currently ongoing, is comparing conventional closure with drains to quilting without drains [17]. The QUILT trial is a multicentre randomised study conducted across nine hospitals in the Netherlands. This comprehensive trial aims to gather detailed data on various outcomes, including the incidence of seroma and postoperative pain, assess shoulder function recovery, and analyse healthcare consumption patterns among patients. By collecting this diverse range of information, the QUILT trial seeks to improve surgical care and postoperative management [18,19].

## Conclusions

Our survey has demonstrated a lack of consensus among UK breast surgeons regarding practices to prevent seroma formation among post-mastectomy patients. At our unit, we have demonstrated a significant reduction in seroma rates and defined a replicable and quick method of quilting, highlighting that it is a safe and effective procedure. Distribution of these results led to an improved perception of quilting as a preventative strategy. There is also increasing interest in conducting studies to standardise practice and inform national guidelines.

## Figures and Tables

**Figure 1:**
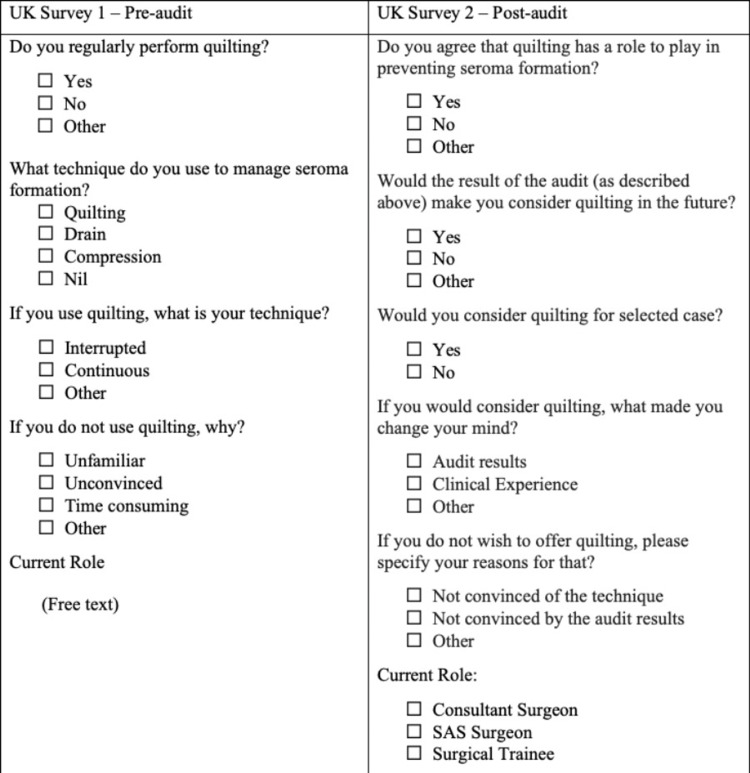
Survey questionnaires used for collecting surgeons' opinion about the quilting technique Survey questionnaires used to collect surgeons' opinions before (Survey 1) and after (Survey 2) sharing the audit results on the quilting technique
